# Models including preoperative plasma levels of angiogenic factors, leptin and IL-8 as potential biomarkers of endometrial cancer

**DOI:** 10.3389/fonc.2022.972131

**Published:** 2022-11-24

**Authors:** Luka Roškar, Maja Pušić, Irena Roškar, Marko Kokol, Boštjan Pirš, Špela Smrkolj, Tea Lanišnik Rižner

**Affiliations:** ^1^ Department of Gynaecology and Obstetrics, Faculty of Medicine, University of Ljubljana, Ljubljana, Slovenia; ^2^ Division of Gynaecology and Obstetrics, General Hospital Murska Sobota, Murska Sobota, Slovenia; ^3^ Institute of Biochemistry and Molecular Genetics, Faculty of Medicine, University of Ljubljana, Ljubljana, Slovenia; ^4^ Faculty of Electrical Engineering and Computer Science, University of Maribor, Maribor, Slovenia; ^5^ Semantika Research, Semantika d.o.o., Maribor, Slovenia; ^6^ Division of Gynaecology and Obstetrics, University Medical Centre, Ljubljana, Slovenia

**Keywords:** angiogenic factors, endometrial cancer, diagnostic biomarkers, angiogenesis, machine learning models, leptin, IL-8, sTie-2

## Abstract

**Background:**

The diversity of endometrial cancer (EC) dictates the need for precise early diagnosis and pre-operative stratification to select treatment options appropriately. Non-invasive biomarkers invaluably assist clinicians in managing patients in daily clinical practice. Currently, there are no validated diagnostic or prognostic biomarkers for EC that could accurately predict the presence and extent of the disease.

**Methods:**

Our study analyzed 202 patients, of whom 91 were diagnosed with EC and 111 were control patients with the benign gynecological disease. Using Luminex xMAP™ multiplexing technology, we measured the pre-operative plasma concentrations of six previously selected angiogenic factors – leptin, IL-8, sTie-2, follistatin, neuropilin-1, and G-CSF. Besides basic statistical methods, we used a machine-learning algorithm to create a robust diagnostic model based on the plasma concentration of tested angiogenic factors.

**Results:**

The plasma levels of leptin were significantly higher in EC patients than in control patients. Leptin was higher in type 1 EC patients versus control patients, and IL-8 was higher in type 2 EC versus control patients, particularly in poorly differentiated endometrioid EC grade 3. IL-8 plasma levels were significantly higher in EC patients with lymphovascular or myometrial invasion. Among univariate models, the model based on leptin reached the best results on both training and test datasets. A combination of age, IL-8, leptin and G-CSF was determined as the most important feature for the multivariate model, with ROC AUC 0.94 on training and 0.81 on the test dataset. The model utilizing a combination of all six AFs, BMI and age reached a ROC AUC of 0.89 on both the training and test dataset, strongly indicating the capability for predicting the risk of EC even on unseen data.

**Conclusion:**

According to our results, measuring plasma concentrations of angiogenic factors could, provided they are confirmed in a multicentre validation study, represent an important supplementary diagnostic tool for early detection and prognostic characterization of EC, which could guide the decision-making regarding the extent of treatment.

## 1 Introduction

Endometrial cancer (EC) is the most frequent gynecological malignancy in developed countries, with an increasing incidence rate (1, 2). The diversity of EC dictates the need for precise early diagnosis and pre-operative stratification to appropriately select the extent of surgery and lower both the recurrence rate and risk for overtreatment. A dualistic model, built mainly on the histological findings and prognosis, was introduced by Bokhman back in 1983 (3–5). Recent advances based on molecular classification stratify EC into four risk categories: POLE ultramutated, microsatellite instability hypermutated, copy-number low, and copy-number high (6, 7). This classification of endometrial cancer has been validated and incorporated in the ESMO/ESTRO risk stratification and is currently used in clinical practice to guide management decisions. However, refinements of the current classification with additional biomarkers are likely to further improve and de-escalate treatment in certain subtypes of EC (8).

Biomarkers represent a noninvasive approach for more precise stratification of various malignant diseases (9). No single serum/plasma biomarker alone has yet been classified for clinical use in the diagnostics of EC (10). Emerging findings in genomics, transcriptomics, and proteomics may present a pivotal role for noninvasive early diagnostic options (11).

Angiogenesis is the process of the formation of new vessels from the preexisting vasculature. The process may be activated due to ischemia and tissue trauma as part of normal tissue healing, or it can be one of the key processes in cancerogenesis, allowing fast growth of cancerous tissue and spreading to distant organs. Tumor tissue receives oxygen and nutrition at its very early stage *via* diffusion independently of the vascular network. When the tumor size exceeds 1–2 mm^3^, the existing capillary network becomes insufficient, causing hypoxia in solid tumors. The resulting shortage of cellular oxygen and nutrients causes the production of angiogenic factors (AFs), mostly cytokine molecules, which are secreted into the surrounding tissue and provoke the growth of new vessels (12–16). This angiogenic switch makes AFs potential biomarker candidates for early EC detection and assessment of prognosis (12, 17, 18).

In our previous research, we investigated 37 AFs as potential biomarkers for EC. AFs’ concentrations in preoperative plasma samples of patients with endometrioid EC (n = 38) and control patients with benign gynecological conditions (e.g., prolapsed uterus or myoma; n = 38) were measured using Luminex xMAP™ multiplexing technology. Our discovery study demonstrated significant differences in the plasma levels of six AFs: sTie-2, G-CSF, and leptin were present in different concentrations in EC versus control patients, and IL-8, neuropilin-1 and follistatin differed among different EC subgroups (19). The roles of these AFs have been described in detail in our recent review paper (12). In the present study, we aimed to validate our previous findings in a larger group of patients.

Recently, machine learning algorithms, a part of artificial intelligence, have evolved to help develop cancer risk stratification systems with great precision. With the aim to differentiate between patient groups, the machine learning approach simultaneously consider multiple disease-specific risk factors, which would otherwise present an impossible statistical obstacle, especially in the heterogeneous type of disease like EC (20, 21). In our study, besides basic statistical methods, we used machine learning algorithms to create a robust diagnostic model based on the plasma concentration of tested AFs.

## 2 Materials and methods

### 2.1 Patient enrollment

Patient enrolment took place between June 2012 and October 2021 at the Department of Obstetrics and Gynecology, University Medical Centre Ljubljana, Slovenia. We included 215 consecutive eligible women who underwent surgical treatment, including a group of histologically confirmed EC patients (n = 98) and a control group of women with a prolapsed uterus or myoma (n = 117). 13 women were excluded from data analysis (7 EC patients and 6 control patients) due to the presence of other malignancies, withdrawal of consent or when surgery was subsequently cancelled for any other reason.

The patients were recruited by senior gynecologists with the help of study nurses. All histological analysis was performed in the University Medical Centre Ljubljana, Department of Pathology. Each sample was consecutively analysed by two pathologists and their consensus report was logged into study case report form. None of the included patients received drugs with known anti-angiogenic effects, and no neoadjuvant chemotherapy was used. One day to one week prior to surgery, morning blood samples were collected, and additional information was obtained regarding patients’ lifestyle, medications used, and gynecological and clinical status (**Table 1**). For sample collection and processing, strict and detailed standard operating procedures were followed. Briefly, 6 ml of blood was collected from each patient by venipuncture using BD vacutainer K2 EDTA tubes (Cat. No#: 367864, BD Medical, New Jersey, USA). Immediately after collection, tubes were inverted 10 times to assure sufficient mixing of blood with anticoagulant. Collected blood samples were centrifuged within 1 hour of collection at 1400 x g for 10 minutes at 4°C. Obtained plasma was transferred to a 5 ml polypropylene tube (Cat. No#: 352063, BD Medical) and mixed several times using a disposable plastic Pasteur pipette. Finally, plasma samples were divided into 200 µl aliquotes and stored in cryogenic tubes (Cat. No#: 375418, Thermo Scientific, Waltham, Massachusetts, USA) at – 80°C until further analysis.

**Table 1:** Detailed clinical characteristics of the study participants.

	Control patients n = 111 (100%)	EC patients n = 91 (100%)	p^a^ values
**Age category**			
<50 years	33 (29.7)	8 (8.8)	
50–59.9 years	38 (34.2)	22 (24.2)	<0.001
60–69.9 years	25 (22.5)	40 (44.0)	
70-79.9 years	14 (12.6)	16 (17.6)	
>80 years	1 (0.9)	5 (5.5)	
**Body mass index (kg/m^2^)**			
<18.5 (underweight)	1 (0.9)	0 (0)	
18.5–24.9 (normal weight)	40 (36.0)	19 (20.9)	
25–29.9 (overweight)	41 (36.9)	25 (27.5)	
30–34.9 (class I obesity)	23 (20.7)	22 (24.2)	<0.001
35–39.9 (class II obesity)	5 (4.5)	13 (14.3)	
40–49.9 (class III obesity)	1 (0.9)	9 (9.9)	
> 50.0 (class IV obesity)	0 (0)	1 (1.1)	
Missing data	0 (0)	2 (2.2)	
**Smoking status**			
Nonsmoker	68 (61.3)	63 (69.2)	
Smoker	21 (18.9)	11 (12.1)	ns
Former smoker	19 (17.1)	15 (16.5)	
Missing data	3 (2.7)	2 (2.2)	
**Hormonal therapy in the past**			
No	67 (60.4)	56 (61.5)	
Yes	9 (8.1)	6 (6.6)	ns
Missing	35 (31.5)	29 (31.9)	
**Peroral contraception in the past**			
No	60 (54.1)	41 (45.1)	
Yes	26 (23.4)	21 (23.1)	ns
Missing	25 (22.5)	29 (31.9)	
**Diabetes**			
No	94 (84.7)	70 (76.9)	
Yes	15 (13.5)	21 (23.1)	ns
Missing data	2 (1.8)	0 (0)	
**Arterial hypertension**			
No	86 (77.5)	49 (53.8)	
Yes	24 (21.6)	42 (46.2)	<0.001
Missing data	1 (0.9)	0 (0)	
**Menopausal status**			
No	46 (41.4)	15 (16.5)	
Yes	62 (55.9)	75 (82.4)	<0.001
Missing data	3 (2.7)	1 (1.1)	

a p values were calculated using non-parametric Mann–Whitney test for continuous variables and chi-squared test for categorical variables; ns = not significant

This study was approved by the National Medical Ethics Committee of the Republic of Slovenia (No. 0120-515/2017/4 and 0120-541/2019/7). All participants signed written informed consent before participating in this study.

### 2.2 Measurements of AFs

All samples were anonymized, and the person performing the assays was blind to the identity of the samples. Plasma samples were tested for 6 circulating angiogenesis biomarkers using Luminex xMAP multiplexing technology with two Milliplex^®^ MAP Human Angiogenesis/Growth Factor Magnetic Bead Panels: HANG2MAG-12K and HAGP1MAG-12K (Merck Millipore, Burlington, Massachusetts, USA, LOT#3601567 and LOT#3601566, respectively). All tests were performed according to the manufacturer’s protocol. Briefly, 10 µl of each plasma sample was used for the conduction of assays. Samples were diluted 1:3 (4 AFs- Bead Panel 1) and 1:5 (2 AFs- Bead Panel 2) using the Assay Buffer provided in the manufacturer’s kit. Samples were mixed with 5.6 µm polystyrene beads on 96 well plates and incubated overnight at 4°C with shaking. Each bead was coated with a specific captured antibody and labelled with two different fluorescent dyes at different ratios assigned for each individual antibody. Plates were washed 3 times and incubated with detection antibody at room temperature (RT) for 1h. Following incubation with streptavidin-phycoerythrin for 30 minutes, plates were washed again, and Drive Fluid was added to all wells. Reading was performed on a MagPix^®^ instrument (Luminex, Austin, Texas, USA). Bio-Plex Manager Software (Bio-Rad Laboratories, Hercules, California, USA) and five-parameter logistic regression modelling were used to calculate the final concentrations.

### 2.3 Statistics

To assess the normality of the distributions, a Shapiro-Wilk test was used. For univariate statistical analysis, the parametric t-test or non-parametric Mann-Whitney U test was used to assess the statistical significance of the difference in plasma concentrations of 6 AF between EC patients and control patients and between different subgroups of EC patients. The non-parametric Kruskal–Wallis test with Dunn’s multiple comparison corrections as *post-hoc* tests was used to compare more than two groups. Fisher’s exact and Chi-square tests were used for comparison of categorical variables. Statistical significance was set at p < 0.05. Results of the descriptive analysis (i.e. patient’s clinical data) were presented as mean ± standard deviation (SD), while the concentrations of the measured proteins were presented as median and range (**Tables 1**, **2**, respectively). Before further analysis, we excluded measurements with reported out-of-range concentrations. Outliers were detected and excluded from further analysis, using the ROUT method (22) with cut off set at 0,1%.

**Table 2:** Plasma leptin and IL-8 levels in patients with endometrial cancer and control patients.

Patient group	n (%)	Leptin		p	IL-8		p
		Median	Range	(adj. p)^a^	Median	Range	(adj. p)^a^
**Disease status**							
EC	91 (45.0)	36654	6248 - 149743	<0.0001	4.16	0.83 – 11.74	0.0619
Benign	111 (55.0)	23121	1526 - 93115	(0.0006)	3.08	0.39 – 10.75	(0.3185)
**Histology**							
Type I	65 (74.7)	37715	6248 – 173402	0.1244	4.16	0.83 – 11.74	0.9594
Type II	22 (25.3)	34015	8183 – 72174	(0.5494)	4.29	0.83 – 12.48	(1.000)
**EC differentiation**							
Well differentiated G1	46 (61.3)	38921	6248 - 173402	0.7636	3.47	0.83 – 9.17	0.0051
Moderately differentiated G2	19 (25.3)	35638	12328 - 149743	(0.9998)	4.70	1.42 – 6.97	(0.0302)
Poorly differentiated G3	10 (13.3)	35581	8183 - 61204		5.69	0.83 – 36.7	
**FIGO stage**							
IA	58 (65.9)	33436	6248 - 109865	0.3607	3.52	0.83 – 6.87	0.0206
IB	12 (13.6)	37568	12384 - 118709	(0.9317)	4.72	2.02 – 13.66	(0.1174)
II	8 (0.09)	54979	8183 - 149743		6.26	1.77 – 12.48	
III	6 (0.07)	40824	34477 - 106658		4.35	2.93 – 8.38	
IV	4 (0.05)	55375	16834 - 59024		12.02	0.83 – 36.70	
**Myometrial invasion**							
No invasion	28 (32.6)	37885	6248 – 173402	0.6168	3.07	0.83 – 5.72	0.0080
< 50% myometrium	32 (37.2)	34727	6661 – 122861	(0.9968)	4.11	1.10 – 11.74	(0.0471)
> 50% myometrium	26 (30.2)	46184	8183 – 149743		4.70	0.83 – 18.37	
**Lymphovascular invasion**							
No	65 (74.7)	35089	6248 – 122861	0.2144	3.52	0.83 – 6.97	0.0004
Yes	22 (25.3)	47372	8183 – 149743	(0.7649)	5.36	0.83 – 18.37	(0.0024)
**Metastasis**							
No	78 (88.6)	35089	6248 - 122861	0.1854	4.16	0.83 – 11.74	0.2595
Yes	10 (11.4)	48380	16834 - 106658	(0.7078)	4.70	0.83 – 18.37	(0.8351)

a p-values were calculated using non-parametric Mann-Whitney tests or Kruskal-Wallis tests with post hoc test and Dunn’s correction. Bonferroni-Šídák method was used for multiple comparison correction and adjusted p-values are listed in parenthesis. Plasma levels of sTie-2, follistatin, neuroplin and G-CSF are included in **Supplementary Table S1**.

### 2.4 Machine learning based classification

The case/control classification models were created using the dataset of the 202 women described in the Patient Enrolment section using the scikit-learn library version 1.0.1 (23) for model training and evaluation. Based on well-performing algorithms for small datasets (24), the ml-jar library version 0.11.2 (25, 26) was chosen to automate the model selection and perform the hyper-parameters tuning and design an ensemble-based classification model.

Preprocessing included the following steps: all out-of-range values below the detection threshold (11.1 pg/ml for follistatin, 5.4 pg/ml for G-CSF, 0.2 pg/ml for IL-8, 42.8 pg/ml for leptin, 12.2 pg/ml for sTie-2 and 54.9 pg/ml for neuropilin-1) were replaced with 0, all out-of-range values above the detection threshold were defined as missing, and finally, missing data imputation was performed. Imputation was done using the mean method for interval/ratio level data and the mode method for categorical data (27).

The dataset was then split into training and test datasets using the scikit-learn library (23), using the built-in train/test split function in an 80% to 20% ratio. All variables were then compared using the Mann-Whitney U statistics to confirm no significant differences between the training and the test datasets. Finally, data were imported into Python using the Pandas library (28, 29), and finally, input and output column selection was performed for each of the hypotheses tested.

The models were trained by optimising the area under the curve (AUC) for the Receiver Operating Characteristic (ROC) curve using the mljar built-in roc_auc metric. The training was performed for 20 minutes for multivariate models and for 15 minutes for univariate models, with a 5-minute limit per individual run. The model selection during training was performed using the k-fold cross-validation method with 20 folds on each model; the threshold for deciding the prediction was calculated based on the ROC curve of the best model by maximising the difference between the true positive rate and the false positive rate on the training set.

The best performing model was then tested using the previously described testing set, again calculating the same basic metrics and validating the models still perform well on previously unseen data. To confirm that the model performance was significantly better than random guessing, Fisher’s Exact test was used on the confusion matrix produced on the training and test datasets.

Two types of models were designed using the described approach: univariate models for each AF and BMI individually and multivariate models for the following combinations:

− Age + BMI + AFs, containing: age, BMI, neuropilin-1, sTie-2, IL-8, follistatin, leptin, G-CSF
− BMI + AFs, containing: BMI, neuropilin-1, sTie-2, IL-8, follistatin, leptin, G-CSF
− AFs only, containing: neuropilin-1, sTie-2, IL-8, follistatin, leptin, G-CSF
− BMI + AFs without leptin: BMI, neuropilin-1, sTie-2, IL-8, follistatin, G-CSF
− AFs only without leptin: neuropilin-1, sTie-2, IL-8, follistatin, G-CSF
− Selected features: age, IL-8, leptin and G-CSF
The “Selected features” model was designed based on the mljar automated feature selection capability, which works in two steps (30):
− A random input feature is created and a model trained on it; SHAP importance (31) is calculated for the feature.
− The model is trained on the rest of the features, and only those features that have a higher SHAP importance than the introduced random feature are used in training. Those features included the aforementioned: age, IL-8, leptin and G-CSF.

Lastly, the feature importance was calculated using the permutation method (32). All models’ output (predicted) variable was whether they belonged to the case or control group. The complete Jupyter Notebook (iPython) and Python scripts used for the model training, validation, plot drawing, and dataset schema are published on: https://github.com/klokedm/EndometrialCancerModelling.

## 3 Results

### 3.1 Characteristics of EC and control patients

The case group included 91 EC patients with a mean age of 62.1 ± 9.8 years (range: 32–86 years) and a mean body mass index (BMI) of 31.2 ± 7.7 kg/m2 (range: 18.8 – 58.5 kg/m2). 75 women (82,4%) were postmenopausal with an average duration of menopause of 12.6 ± 8.4 years. The detailed clinical characteristics are presented in **Table 1**.

Histology revealed 76 cases (83.5%) of endometrioid endometrial cancer (EEC), 9 cases (9.9%) of serous EC and one case (1.1%) of each of carcinosarcoma, clear cell EC and mixed type EC. The deep myometrial invasion was observed in 26 EC patients (30.2%), <50% invasion into the myometrium in 32 EC patients (37.2%), and no invasion into the myometrium in 28 EC patients (32.6%); this information was missing for five patients. LVI was observed in 22 patients (25.3%). According to the classification of the International Federation of Gynecology and Obstetrics (33), the following EC stages were observed: IA (n = 58, 65.9%), IB (n = 12, 13.6%), II (n = 8, 9.1%), III (n = 6, 6.8%), and IV (n = 4, 4.5%). Histopathological evaluation divided EC samples according to the degree of histological differentiation: G1, 47 cases; G2, 20 cases; and G3, 12 cases.

The control group included 111 patients with a mean age of 56.5 ± 10.5 years (range: 37–84 years) and a mean BMI of 27.0 ± 4.9 kg/m2 (range: 18.4–42.2 kg/m2). 62 women (55.9%) were postmenopausal, with an average duration of menopause of 12.7 ± 8.5 years. The detailed clinical characteristics are presented in **Table 1**.

When both groups were compared, there was a statistically significant difference in BMI and age distribution, as well as in menopausal status and in the presence of arterial hypertension (all p < 0.001). There were no differences between groups in hormonal therapy, smoking status or the presence of diabetes (**Table 1**).

### 3.2 Leptin is increased in EC patients and IL-8 in patients with LVI and MI

**Figure 1:**
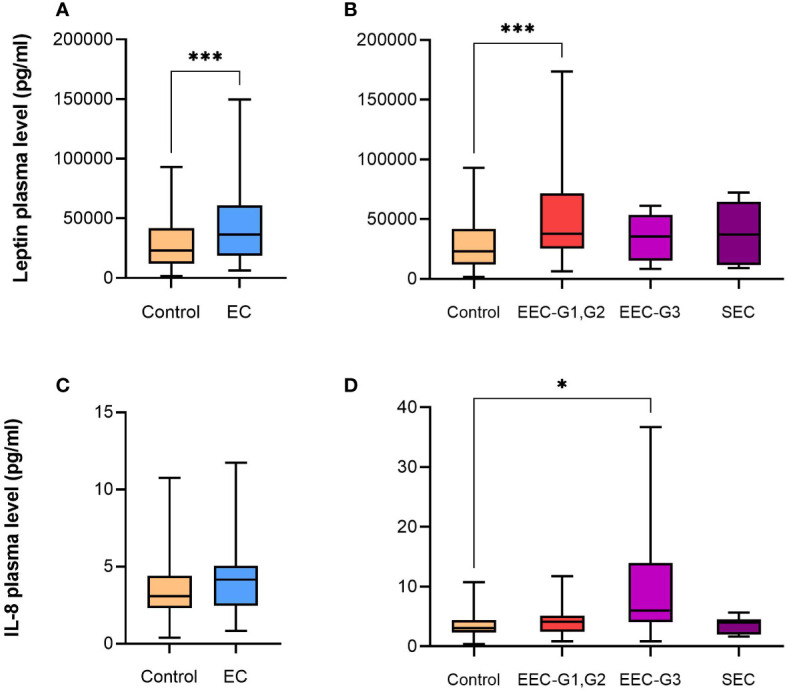
Box plots with median, minimum and maximum for plasma leptin and IL-8 levels (pg/mL). **(A, C)** Control patients and patients with endometrial cancer. **(B, D)** Control patients and patients with histological subtypes of endometrial cancer – Type I (EEC G1, G2) and Type II (EEC G3 and SEC). EC – endometrial cancer, EEC – endometrioid endometrial cancer, SEC – serous endometrial cancer, G – grade. Adjusted p values: ^*^p < 0.05, ^***^p < 0.001.

**Figure 2:**
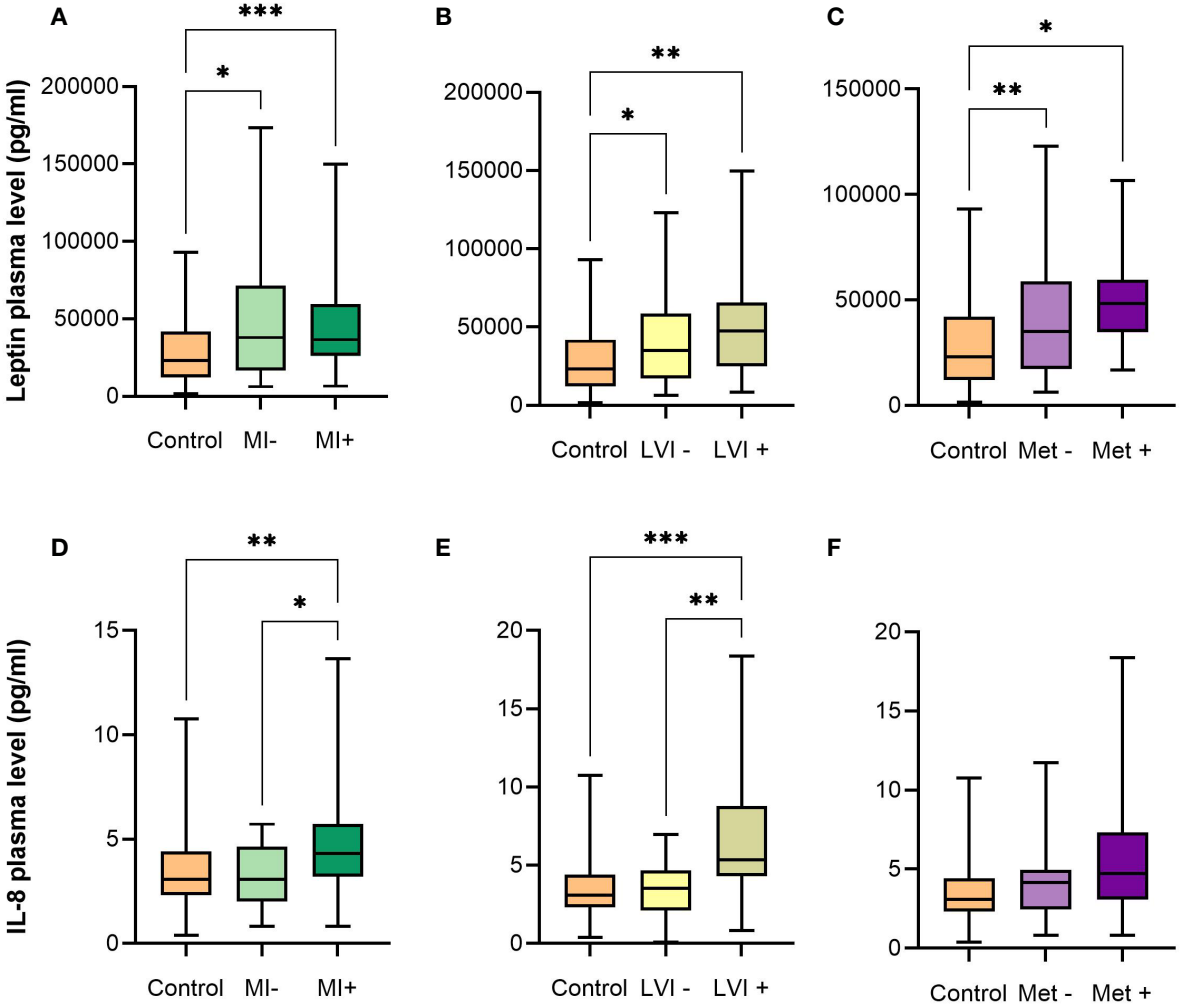
Box plots with median, minimum and maximum for plasma leptin and IL-8 levels (pg/mL). **(A, D)** Control patients and EC patients with absence or presence of myometrial invasion. **(B, E)** Control patients and EC patients with absence or presence of lymphovascular invasion. **(C, F)** Control patients and EC patients with absence or presence of metastasis. EC – endometrial cancer, MI – myometrial invasion, LVI – lymphovascular invasion, Met – metastasis. Adjusted p values: ^*^p < 0.05, ^**^p < 0.01, ^***^p < 0.001.

Univariate statistical analysis revealed a significant difference in the plasma concentration of leptin between EC and control patients (**Figure 1**; **Table 2**). The difference in IL-8 levels between EC and the control group was also substantial, although not statistically significant. IL-8 was significantly higher in patients with poorly differentiated G3 EC and in EC patients with present myometrial (MI) or lymphovascular invasion (LVI) compared to EC patients without invasion or control patients. Compared to control patients, leptin was significantly higher in EC patients regardless of MI or LVI or the presence of metastasis (**Figure 2**).

**Figure 3:**
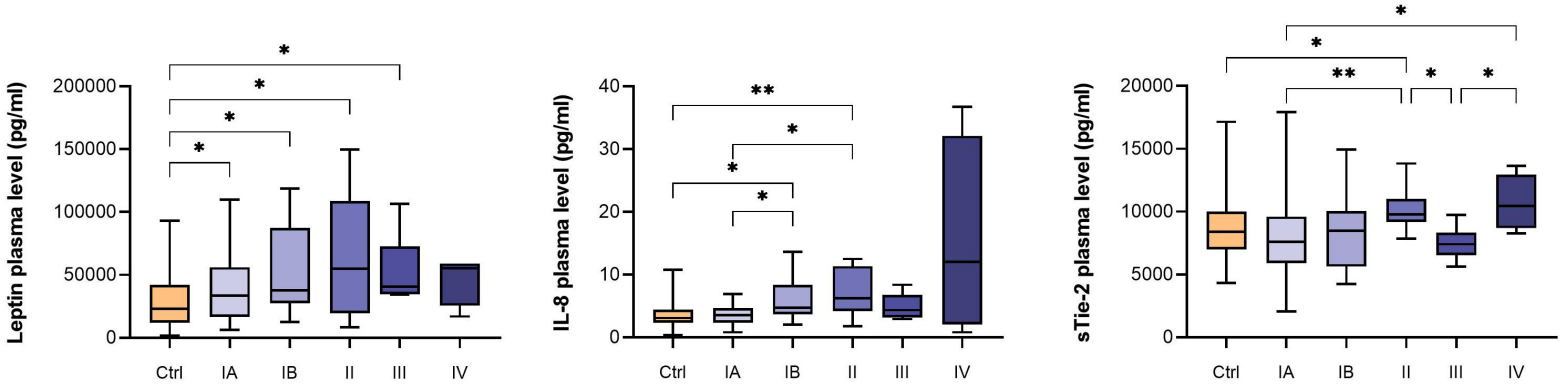
Box plots with median, minimum and maximum for plasma leptin, IL-8 and sTie-2 levels (pg/mL) for control patients and EC patients in different FIGO stages. P-values were calculated using Kruskal-Wallis tests with *post hoc* test without correction. *p < 0.05; **p < 0.01.

When EC patients were stratified according to the disease stages, we detected higher plasma levels of leptin, IL-8 and sTie-2 as EC progressed from stage IA through stage II (**Figure 3**, **Table 2** and **Supplementary Table 1**). This trend was not seen in stages III and IV, which might be due to a low number of patients in these stages (6 and 4 patients, respectively). Leptin was also significantly higher in type 1 EC patients versus control patients, whereas IL-8 was higher in type 2 EC versus control patients, particularly in poorly differentiated endometrioid EC grade 3 (**Figure 1**; **Table 2**).

The prognostic potential of AFs was evaluated based on the presence of MI and LVI. There was a statistically significant difference in IL-8 plasma levels between patients with or without MI and LVI (**Figure 2**). Although IL-8 is also produced and secreted by adipocytes, and its levels increase with BMI, which is known to be associated with EC incidence and outcomes (34), we found no correlation between IL-8 and BMI with Pearson correlation coefficient of 0.0009 (**Suplementary Table 8**). Plasma levels of IL-8 were able to stratify EC patients according to the MI (p = 0.0057) and LVI (p = 0.0005), whereas BMI, which is not used in clinical practice to asses EC risk, was not successful in differentiation among groups: p = 0.8669 and 0.6156 for MI and LVI, respectively. The data is presented in **Supplementary Figure 1**.

### 3.3 Machine learning approaches identified several diagnostic models

The training dataset contained 161 patients (79.7%) and the testing dataset contained 41 patients (20.3%), and none of the 8 variables used in modelling were significantly different between the training and test datasets with p-values for: age (p=0.495), BMI (p=0.721), neuropilin-1 (p=0.277), sTie-2 (p=0.826), IL-8 (p=0.556), follistatin (p=0.785), leptin (p=0.848), G-CSF (p=0.256).

The AFs in the complete data set were weakly correlated (correlation coefficient < 0.3) with known risk factors (Age and BMI), with the exception of BMI and Leptin, which were strongly correlated (correlation coefficient > 0.7). All correlations are shown in **Supplementary Table 8**.

#### 3.3.1. Model based on leptin performed significantly better than random guessing

The results obtained on the final ensemble using the training dataset (best metric for each metric in bold) are shown in **Table 3**. The table presents the Accuracy, Precision, F1 score and Receiver Operating Characteristic (ROC) Area Under Curve (AUC) metrics for all seven univariate models calculated on the training dataset using the metrics presented in **Supplementary Table 2**. The models with the highest accuracy were based on leptin and IL-8 on the training dataset, the model with the highest precision was based on follistatin, and the model with the highest F1 score and ROC AUC was based on leptin. The model based on leptin reached the best result on 3 out of 4 metrics: accuracy, F1 score and ROC AUC.

**Table 3:** Performance metrics of univariate models on the training dataset based on single angiogenic factors.

Model	Accuracy	Precision	F1 score	ROC AUC
*BMI*	0.66	0.65	0.59	0.68
*Neuropilin-1*	0.54	0.50	0.62	0.55
*sTie-2*	0.65	0.60	0.65	0.68
*IL-8*	**0.75**	0.74	0.71	0.83
*Follistatin*	0.73	**0.75**	0.67	0.77
*Leptin*	**0.75**	0.69	**0.74**	**0.80**
*G-CSF*	0.61	0.58	0.56	0.63

Best metric shown in bold.

The results obtained on the final ensemble using the test dataset (best metric for each model in bold) are shown in **Table 4**. The table presents the Accuracy, Precision, F1 score and Area Under Curve (AUC) metrics for all seven univariate models calculated on the test dataset using the metrics presented in **Supplementary Table 3**. The model based on leptin reached the best result on all 4 metrics: accuracy, precision, F1 score and ROC AUC, and outperformed other AFs as well as BMI as an EC risk predictor.

**Table 4:** Performance metrics of univariate models on the test dataset based on single angiogenic factors.

Model	Accuracy	Precision	F1 score	ROC AUC
*BMI*	0.57	0.50	0.56	0.57
*Neuropilin-1*	0.35	0.33	0.40	0.39
*sTie-2*	0.41	0.31	0.31	0.50
*IL-8*	0.59	0.52	0.59	0.61
*Follistatin*	0.62	0.57	0.53	0.54
*Leptin*	**0.70**	**0.61**	**0.72**	**0.75**
*G-CSF*	0.59	0.52	0.62	0.55

Best metric shown in bold.

**Figure 4:**
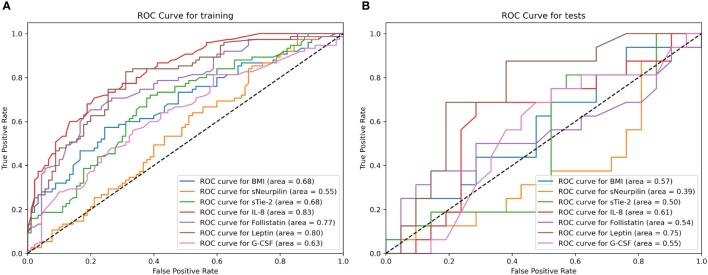
The Receiver Operating Characteristic (ROC) curve for all seven final univariate models on the training **(A)** and test **(B)** dataset calculated using the cross-folding method, as described in Section 2.4. **(A)** The curve shows how the true positive rates of individual models change according to the false positive rate, by varying the detection threshold in the model. The AUC metric for each curve is displayed in the parentheses. As can be seen, all models except neurpilin-1 perform better than random chance on the training dataset and the models are able to discern between case and control group with an AUC ≥ 0.63. **(B)** The curve shows how the true positive rates of individual models change according to the false positive rate by varying the detection threshold in the model. The AUC metric for each curve is displayed in the parentheses. As seen from the curve, only leptin and IL-8 based models deviate from the random line, and as shown in the performance metrics, only leptin reaches a statistically significant accuracy at the optimal ROC point. This indicates that the models (with the exception of the mentioned ones) overfit on the training data.

Additionally, the Fisher Exact p-values calculated for confusion matrices on the test datasets on univariate models are shown in **Supplementary Table 4**. They show that all models, except the model based on neuropilin-1, performed significantly better than random guessing on the training dataset. However, only the model based on leptin performed significantly better than random guessing on both the training and the test datasets with a significance level of p < 0.01. The ROC curves for all seven final univariate models on the training and test datasets are shown in **Figure 4**.

#### 3.3.2. The model, including the combination of all AFs, BMI and age, shows the best diagnostic characteristics

The results obtained on the final ensemble using the training dataset (best model for each metric in bold) are shown in **Table 5**. They show that the best results for all metrics were obtained for the model utilizing the subset of features determined using the method described in the Methodology section. However, all models achieved good results on all metrics, and discrepancies were minimal (**Supplementary Table 5**).

**Table 5:** Performance metrics of multivariate models based on multiple features on training data.

Model	Accuracy	Precision	F1 score	ROC AUC
*Age+BMI+AFs*	0.85	0.83	0.83	0.89
*BMI + AFs*	0.82	0.84	0.77	0.86
*AFs*	0.85	0.83	0.83	0.89
*BMI + AFs without Leptin*	0.84	0.79	0.83	0.92
*AFs without Leptin*	0.81	0.75	0.79	0.86
*Selected Features*	**0.86**	**0.85**	**0.84**	**0.94**

Best metric shown in bold.

**Figure 5:**
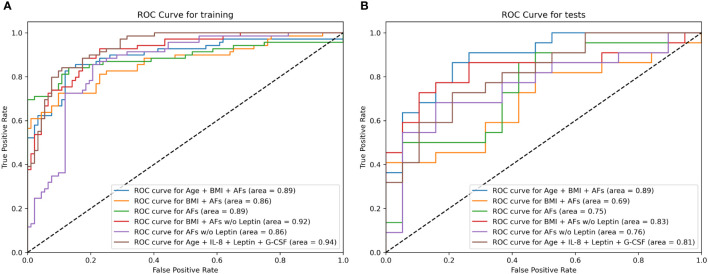
Receiver Operating Characteristic (ROC) curve for the six final multivariate models on the training **(A)** and test **(B)** dataset calculated using the cross-folding method, as described in Section 2.4. **(A)** The curve shows that all multivariate models were able to discern between case and control group on the training dataset with relatively good results (AUC > 0.85). **(B)** Most models utilizing multiple features were able to generalize to unseen data and maintained a relatively robust classification performance with ROC AUC > 0.80. The model utilizing a combination of all AFs, BMI and age reached an ROC AUC of 0.89 on both the training and test dataset.

The results obtained on the final ensemble using the test dataset (best model for each model in bold) are presented in **Table 6** (**Supplementary Table 6**) – the discrepancies are bigger on the test data in line with the expectations. However, metrics remain good, with the lowest accuracy being 63% and the highest accuracy being 80%, and lowest precision being 73% and the highest precision reaching 100%. The ROC AUC was in the range of 0.69 to 0.89 (**Figure 5**), indicating good model generalization to unseen data.

**Table 6:** Performance metrics of multivariate models based on multiple features on test data.

Model	Accuracy	Precision	F1 score	ROC AUC
*Age+BMI+AFs*	0.76	0.83	0.75	**0.89**
*BMI + AFs*	0.68	**1.00**	0.58	0.69
*AFs*	0.63	0.73	0.59	0.75
*BMI + AFs without Leptin*	**0.80**	0.89	**0.80**	0.83
*AFs without Leptin*	0.71	0.81	0.68	0.76
*Selected Features*	0.76	0.80	0.76	0.81

Best metric shown in bold.

The “Selected Features” model was designed by automatically selecting the features with the estimated highest capability to discriminate between EC and control patients based on SHAP importance. Those features were: age, IL-8, leptin and G-CSF. The corresponding model reached a ROC AUC of 0.94 on the training dataset and 0.81 on the test dataset. The model utilizing a combination of all AFs, BMI and age reached a ROC AUC of 0.89 on both the training and test dataset.

The Fisher Exact test analysis results on the test dataset for the multivariate models are presented in **Supplementary Table 7**. Similarly to the univariate models, all models performed significantly better than random chance on the training dataset, and all models where AFs were used together with additional clinical data also performed better than random guessing on the test dataset. Based on this performance, the models were able to generalize the patterns from the training data and could be useful for predicting the risk of EC on unseen data.

## 4 Discussion

Non-invasive diagnostic approaches invaluably assist clinicians in the detection, management and treatment of patients in daily clinical practice. Recently a proof-of-principle was provided in the area of cytological analysis for EC screening: O’Flynn et al. demontrated that endometrial cancer can be detected with high accuracy in urine and vaginal fluid (35). However, there are currently no validated diagnostic or prognostic biomarkers for EC that could accurately predict the presence and extent of disease, and thus pathohistological examination remains the gold standard for EC diagnosis.

In our previous discovery study, we investigated 37 AFs as potential biomarkers for EC. Preoperative plasma samples of 38 EC patients and 38 control patients were analyzed using Luminex xMAP™ multiplexing technology. Six out of 37 AFs were present in significantly different concentrations between groups of patients. sTie-2 and G-CSF were lower in EC compared to control patients, and plasma level of leptin was higher in EC patients. Neuropilin-1 plasma level was higher in patients with type 2 EC (G3) compared to patients with lower grade cancer or controls. Follistatin level was higher in patients with LVI, and IL-8 plasma level was higher in patients with metastases (19). In this validation study, we further evaluated those six AFs on a larger group of patients. While our previous study was limited to post-menopausal women with endometrioid EC, in the present validation study, women with other EC types were also included, regardless of their menopausal status. In the previous study, where 60.5% of included EC patients were in IA stage EC, and no patients in stage II or IV were included, we detected lower values of sTie-2 in EC patients compared to the control group. In the current validation study, we observed a similar trend in the early stages, whereas the concentration of sTie-2 significantly increased as the disease progressed, particularly in FIGO stage IV. This is in accordance with results from Saito et al., who reported higher expression of Tie-2 in endometrial adenocarcinoma than in normal epithelial cells (36).

EC often involves patients burdened with other comorbidities, such as hypertension, diabetes or obesity (37). In our study, diabetes status was balanced between EC and control patients, and only leptin differed among patients when stratified according to hypertension status or obesity. The effect of BMI difference between groups was intensely further investigated through machine learning modelling.

Obesity is an established risk factor for EC and presents a larger risk for this malignancy than any other cancer type (38–41). Adipose tissue is an endocrine organ which synthesizes adipokines—biologically active substances participating in cell growth and differentiation, apoptosis, angiogenesis, and carcinogenesis. Leptin is among the most important adipokines during EC development, and numerous recent studies have already proposed it as a new candidate marker in determining the potential risk of EC (42–45). In accordance with the literature (46–50), our study showed higher leptin levels in EC patients, particularly in Type I EC, at higher stages and in patients with present metastasis or MI and LVI.

During carcinogenesis, leptin promotes tumor angiogenesis. Early studies showed that endothelial leptin receptor Ob-R generates a growth signal involving a tyrosine kinase-dependent intracellular pathway, promoting angiogenic processes (51, 52). Furthermore, leptin induces proliferation, migration, and invasion and suppresses apoptosis of cancer cells (42, 53). However, cancerogenic potential of leptin has been shown to differ between different tissues (54, 55). In EC, it has been shown in *in vitro* studies that leptin inhibits the apoptosis of endometrial carcinoma cells through activation of the nuclear factor κB-inducing kinase/IκB kinase pathway (49) and stimulates endometrial carcinoma cell proliferation *via* enhancing P450arom expression and estradiol synthesis (56).

Leptin has an important role in the regulation of energy balance and glucose metabolism and is considered to play an important part in the link between obesity and EC (57). There is still an ongoing debate in the literature on whether the effect of leptin on EC risk is related to higher BMI or whether it is an independent risk factor for EC. Wang et al. (58) performed a meta-analysis of 6 preceding studies and found that after adjusting for BMI, leptin was still associated with an increased risk of EC. On the other hand, another meta-analysis performed by Ellis et al. observed that BMI and diabetes appeared to affect the association between leptin levels and EC risk (43). Some other studies found a strong positive correlation between patients’ BMI and serum leptin levels (46, 50), while a recent Mendelian randomization analysis showed a causal effect of BMI on EC but failed to find evidence for leptin to be causally implicated in EC risk (59).

By utilizing an automated machine learning approach and comparing univariate models applying BMI and leptin as predictor variables, our research indicates that leptin might be able to predict EC better than BMI. This would seem to be further indicated by the results of the automated feature prediction selection described in the methodology, which identified a combination of age, IL-8, leptin and G-CSF, but not BMI, as the most important features for multivariate model building and the performance of the corresponding model utilizing those features. This supports findings that leptin might be involved in EC development *via* pathways beyond obesity-related pathophysiology, including through angiogenesis (51, 52).

However, as always with machine learning models, this should be interpreted with care. Due to the relatively small data set, we observed overfitting on univariate models, and classification close to random on the test dataset could simply mean the model picked features that were not well generalizable. On the other hand, leptin-based multivariate models performed significantly better than random chance on the test dataset with an AUC very similar to the one reached on the training dataset. This indicates that the models were able to generalize and could be useful for predicting the risk of EC even on unseen data. Therefore, these models show good diagnostic characteristics, with AUC in the range from 0.69 to 0.89.

Another link between adipose tissue and EC is through chronic low-grade inflammation resulting from increased secretion of pro-inflammatory molecules such as cytokines and chemokines (60). IL-8 is a pro-inflammatory cytokine secreted by adipocytes, with well-defined functions in tumor-associated inflammation. It is chemotactic for lymphocytes and neutrophils and also has an important role in angiogenesis (61, 62). Its levels increase with BMI and waist circumference, which is also associated with EC incidence and outcomes (34, 63). Kotowicz et al. (64) demonstrated the clinical usefulness of IL-8 measurements as potential prognostic factors in type 1 EC, where elevated pretreatment IL-8 serum levels were independently associated with shorter disease-free and overall survival.

While in our study, leptin was significantly increased in type I EC, IL-8 was higher in type II EC. Its level also increased through higher grades, and it was particularly elevated in poorly differentiated EEC G3, however the difference between G1/G2 and G3 cases was not statistically significant. We assessed EEC G3 cancers together with type II tumors since several important reports have strongly demonstrated that high-grade endometrioid cancers have molecular characteristics, risk factors, clinical behavior, and prognosis overlapping with those of non-endometrioid cancers (65, 66). We demonstrated that higher levels of IL-8 are suspect for higher-grade tumors. However, there were only 10 G3 EC patients in our cohort. If this result could be validated on a larger set of patients, a simple blood test for IL-8 prior to the surgery might lead to a better stratification for the extent of surgery.

The angiogenic switch occurs early in the process of cancerogenesis. Correspondingly, we observed indicated trend for leptin, sTie-2 and IL-8 progression through early EC stages, which illuminates how growing tumor mass dictates a higher need for additional oxygen and nutrient supply and accelerates angiogenic activity. Nevertheless, due to low number of cases in each EC stage, this should be taken with care and studies on bigger cohort are needed to confirm this.

We also showed a statistically significant correlation between EC myometrial invasion and IL-8 levels. This was previously reported by Fujimoto et al., who suggested IL-8 might act as an angiogenic switch in myometrial invasion in stage I EC (67). According to the currently valid guidelines (10), LVI, among other histopathological characteristics, is the cornerstone of risk stratification. In our study, IL-8 levels significantly increased during LVI, which further demonstrates the role of IL-8 in the angiogenesis of EC. Since IL-8 is also associated with obesity, the prognostic value of IL-8 was compared to BMI based on the presence of MI and LVI. Plasma IL-8 level was a better prognostic biomarker than BMI in terms of EC patient stratification according to both MI and LVI. Levels of IL-8 were also higher in patients with present metastasis, which is in accordance with our previous results (19) and *in vitro* study on cell lines (68).

Due to the relatively low number of patients with LVI and MI in our dataset, the IL-8 link to MI and LVI could not be analysed using the machine learning approach; however, the data presented indicate that it would be a good candidate for a larger study with larger and more balanced datasets.

In our study, we also evaluated neuropilin-1, follistatin and G-CSF as potential biomarkers for EC. While they were individually able to differentiate between EC patients and control patients (with the exception of neuropilin-1), the univariate models utilizing those AFs did not generalize well to unseen (test) data (so-called overfitting). However, when used in conjunction with other data, the AFs significantly improved the classification capabilities of models, and the model utilizing a combination of all AFs, BMI and age reached a ROC AUC of 0.89 on both the training and test dataset, strongly indicating the usefulness of the combination of AFs.

The best multivariate model on the training set (“Selected Features”) has also proven to be robust on both the training and test datasets, where it revealed good diagnostic characteristics with the ROC AUC of 0.94 and 0.81, respectively. It also greatly outperformed individual results of univariate models for included AFs, i.e. leptin, IL-8 and G-CSF.

Our study has also confirmed the importance of existing, well-known risk factors, namely age and BMI, and the values for both were significantly different between the EC and control patients (69, 70). To confirm AFs can valuably add to diagnostics of EC, we created and tested models using only the variables that are – at most – weakly correlated with known risk factors. Using those models, we have confirmed that even without knowing age, BMI or hypertension status, we can reach relatively robust results using only AFs (71% accuracy on the test dataset using AFs without leptin). Nevertheless, combining AFs with the existing risk factors yielded better results (80% accuracy on the test dataset), which confirms the value of already established risk factors in extended models.

Finally, the fact that models utilizing less data sometimes outperformed models utilizing more data might seem counterintuitive but would seem to hint at some combinations of factors containing more noise and thus causing overfitting. In our study, due to noise generation, models using a combination of both BMI and leptin performed weaker than models with either BMI or leptin alone, indicating some collinearity between them. Consequently, a larger study providing more data would be useful to fine-tune the developed diagnostic models and determine the minimal subset required to achieve good classification results.

Our study has the following limitations. First, relatively low number of patients in different EC subgroups – in relation to presence of LVI and MI, EC stage and grade, and EC subtypes. Second, the control group was chosen randomly among women with a prolapsed uterus or myoma who were admitted for surgical procedure, whereas ideal control group for endometrial cancer biomarker discovery would be symptomatic post-menopausal women. In our study patients in control group were relatively younger and with less additional comorbidities than patients with EC. Since these limitations may affect the generalisability of the study conclusions, the results of this study should be validated on bigger cohort in a multicentric clinical study.

To conclude, AFs – especially leptin and IL-8, represent valuable biomarkers candidates with a potential for early diagnostics and risk stratification of EC. Leptin represents a candidate for a diagnostic biomarker of EC, while IL-8 might be valuable in EC patient stratification according to prognostic characteristics, e.g. LVI, MI and EC grade. Other AFs further increased the performance of multivariate models. As revealed through the machine learning approach, the plasma concentrations of AFs, in conjunction with other clinical data, show good diagnostic characteristics. They could, provided they are confirmed in a large-scale multicentre validation study, represent a valuable supplementary diagnostic tool for EC’s early detection and prognostic characterization. This could guide the decision-making regarding the extent of surgery and the choice of adjuvant therapy for EC.

## Data availability statement

The raw data supporting the conclusions of this article will be made available by the authors, without undue reservation.

## Ethics statement

The studies involving human participants were reviewed and approved by the National Medical Ethics Committee of the Republic of Slovenia (No. 0120-515/2017/4 and 0120-541/2019/7. The patients/participants provided their written informed consent to participate in this study.

## Author contributions

LR: Conceptualisation, Data curation, Writing, Result Analysis, Discussion. MP: Methodology; Data analysis, Data curation. IR: Data curation, Writing, review & editing, Statistics, Modelling. MK: Data curation, Statistics, ML Modelling, Results, Writing – review & editing. BP: Methodology; Data curation. ŠS: Conceptualisation; Writing –review & editing; Resources; Supervision. TR: Conceptualisation; Writing – review & editing; Resources; Supervision. All authors contributed to the article and approved the submitted version.

## Funding

This research was funded by two grants: the Slovenian Research Agency grants J3-2535 to TLR; and University Medical Centre Ljubljana tertiary project TP 202110160 to ŠS.

## Acknowledgments

The authors would like to thank study participants who kindly donated their samples and time. We also thank the personnel from the Division of Gynecology and Obstetrics, University Medical Centre, Ljubljana, especially Milena Osredkar, Dr. Leon Meglič, MD, and Vesna Sekelj Rangus, for their help and support in enrolling the study participants and sample collection. We thank Vera Troha Poljančič and Prof. Dr. Joško Osredkar, from the Clinical Institute of Clinical Chemistry and Biochemistry of the University Clinical Centre Ljubljana, for processing and storing the samples according to our standard operating procedures.

## Conflict of interest

The authors declare that the research was conducted in the absence of any commercial or financial relationships that could be construed as a potential conflict of interest.

## Publisher’s note

All claims expressed in this article are solely those of the authors and do not necessarily represent those of their affiliated organizations, or those of the publisher, the editors and the reviewers. Any product that may be evaluated in this article, or claim that may be made by its manufacturer, is not guaranteed or endorsed by the publisher.
