# Supplementary material for: Models including preoperative plasma levels of angiogenic factors, leptin and IL-8 as potential biomarkers of endometrial cancer
Source: Front Oncol. 2022 Nov 24;12:972131. doi: 10.3389/fonc.2022.972131 (PMC9730274; doi:10.3389/fonc.2022.972131)
Supplement: Supplementary file 1 [file DataSheet_1.docx]

Supplementary Material

Models including preoperative plasma levels of angiogenic factors, leptin and IL-8 as potential biomarkers of endometrial cancer

Luka Roškar^1,2^, Maja Pušić^3^, Irena Roškar^3^, Marko Kokol^4,5^, Boštjan Pirš^6^, Špela Smrkolj^1,6*^, Tea Lanišnik Rižner^3*^

^1^ Department of Gynaecology and Obstetrics, Faculty of Medicine, University of Ljubljana, 1000 Ljubljana, Slovenia

^2^ Division of Gynaecology and Obstetrics, General Hospital Murska Sobota, 9000 Murska Sobota, Slovenia

^3^ Institute of Biochemistry and Molecular Genetics, Faculty of Medicine, University of Ljubljana, 1000 Ljubljana, Slovenia

^4^ Faculty of Electrical Engineering and Computer Science, University of Maribor, 2000 Maribor, Slovenia

^5^ Semantika Research, Semantika d.o.o., 2000 Maribor, Slovenia

^6^ Division of Gynaecology and Obstetrics, University Medical Centre, 1000 Ljubljana, Slovenia

*** Correspondence:**Špela Smrkolj, [spela.smrkolj@mf.uni-lj.si](mailto:spela.smrkolj@mf.uni-lj.si)

Tea Lanišnik Rižner, [tea.lanisnik-rizner@mf.uni-lj.si](mailto:tea.lanisnik-rizner@mf.uni-lj.si)


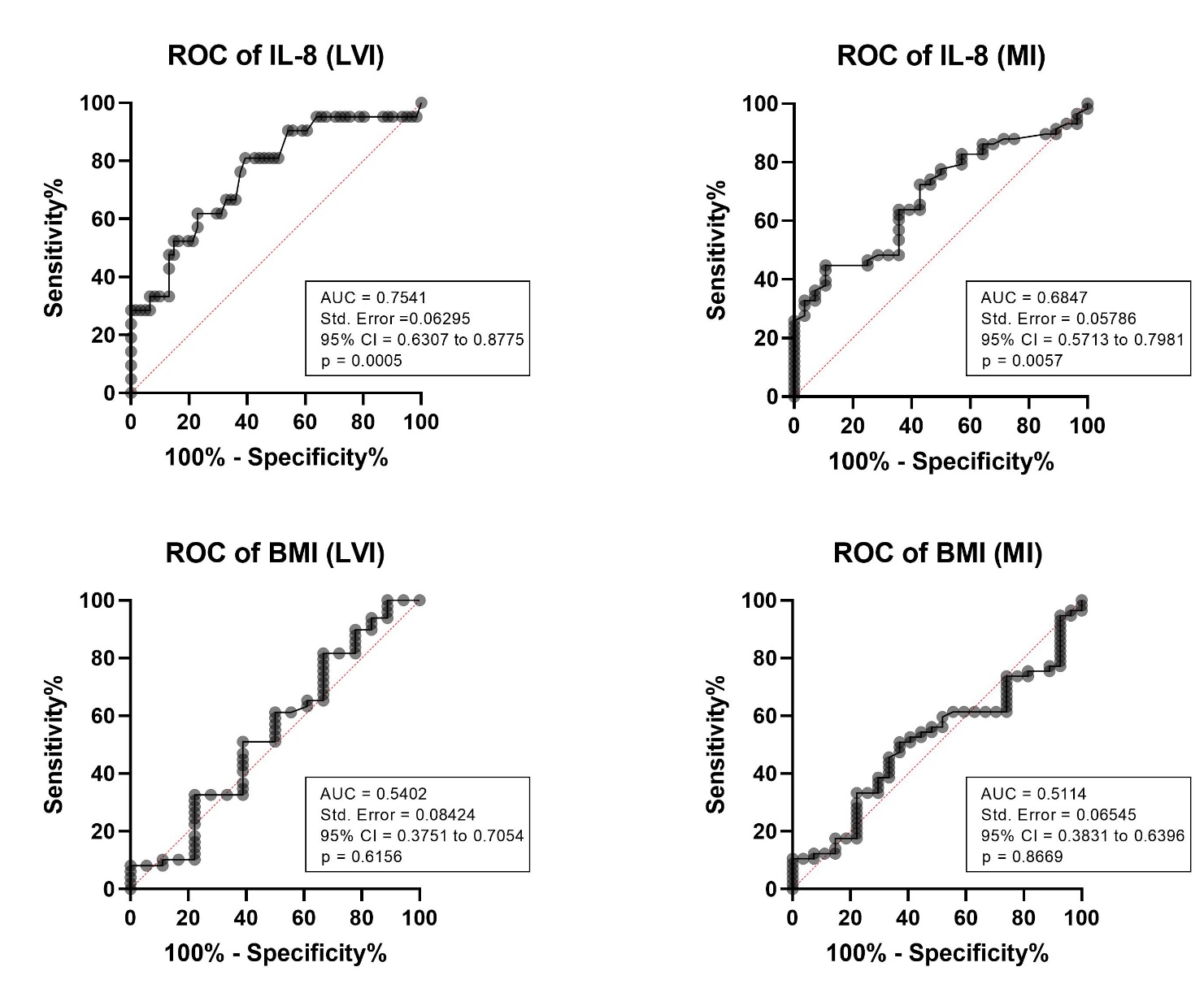


**Supplementary Figure 1:** Prognostic ROC curves, based on the evaluation of myometrial and lymphovascular invasion.

**Supplementary Table 1:** Plasma levels of sTie-2, follistatin, neuroplin and G-CSF in patients with endometrial cancer and control patients.

| Patient group | n (%) | sTie-2 | | p | G-CSF | | p |
| --- | --- | --- | --- | --- | --- | --- | --- |
|  |  | Median | Range | (adj. p)^a^ | Median | Range | (adj. p)^a^ |
| **Disease status** |  |  |  |  |  |  |  |
| EC | 91 (45.0) | 7987 | 2059 - 17931 | 0.3192 | 71.24 | 4.83 – 259.0 | 0.3825 |
| Benign | 111 (55.0) | 8387 | 4322 - 17143 | (0.9004) | 70.44 | 4.83 – 178.0 | (0.9446) |
|  |  |  |  |  |  |  |  |
| **Histology** |  |  |  |  |  |  |  |
| Type I | 65 (74.7) | 7987 | 2391 – 17931 | 0.7217 | 70.44 | 17.77 – 178.0 | 0.5596 |
| Type II | 22 (25.3) | 8083 | 2059 - 13621 | (0.9995) | 69.90 | 4.83 – 116.1 | (0.9927) |
|  |  |  |  |  |  |  |  |
| **EC differentiation** | |  |  |  |  |  |  |
| Well differentiated G1 | 46 (61.3) | 7988 | 2391 – 17931 | 0.4900 | 70.44 | 17.77 – 178.0 | 0.6122 |
| Moderately differentiated G2 | 19 (25.3) | 8019 | 5071 – 14948 | (0.9824) | 70.44 | 29.38 – 266.5 | (0.9966) |
| Poorly differentiated G3 | 10 (13.3) | 8071 | 5491 – 13621 |  | 66.19 | 4.83 – 246.7 |  |
|  |  |  |  |  |  |  |  |
| **FIGO stage** |  |  |  |  |  |  |  |
| IA | 58 (65.9) | 7587 | 2059 – 17931 | 0.0373 | 70.44 | 17.77 – 149.8 | 0.7835 |
| IB | 12 (13.6) | 8497 | 4234 – 14948 | (0.2039) | 80.49 | 29.38 – 178.0 | (0.9999) |
| II | 8 (0.09) | 9798 | 7863 – 13833 |  | 62.48 | 4.83 – 266.5 |  |
| III | 6 (0.07) | 7406 | 5629 – 9731 |  | 61.21 | 50.94 – 100.2 |  |
| IV | 4 (0.05) | 10456 | 8280 – 13621 |  | 70.17 | 34.93 – 246.7 |  |
|  |  |  |  |  |  |  |  |
| **Myometrial invasion** |  |  |  |  |  |  |  |
| No invasion | 28 (32.6) | 7988 | 2059 – 17931 | 0.0287 | 70.44 | 17.77 – 132.8 | 0.8271 |
| < 50% myometrium | 32 (37.2) | 6907 | 4685 – 16860 | (0.1603) | 70.44 | 18.00 – 149.8 | (1.0000) |
| > 50% myometrium | 26 (30.2) | 9418 | 4234 – 14948 |  | 69.33 | 4.83 – 178.0 |  |
|  |  |  |  |  |  |  |  |
| **Lymphovascular invasion** |  |  |  |  |  |  |  |
| No | 47 (72.3) | 7800 | 2059 – 17931 | 0.0510 | 69.60 | 17.77 – 132.8 | 0.7015 |
| Yes | 18 (27.7) | 9418 | 4234 – 16860 | (0.2695) | 70.44 | 4.83 – 266.5 | (0.9993) |
|  |  |  |  |  |  |  |  |
| **Metastasis** |  |  |  |  |  |  |  |
| No | 78 (88.6) | 7988 | 2059 – 17931 | 0.5770 | 70.44 | 4.83 – 178.0 | 0.6024 |
| Yes | 10 (11.4) | 8063 | 5629 – 13621 | (0.9943) | 65.55 | 34.93 – 100.2 | (0.9960) |

^a^ p-values were calculated using non-parametric Mann-Whitney tests or Kruskal-Wallis tests with post hoc test and Dunn’s correction. Bonferroni-Šídák method was used for multiple comparison correction and adjusted p-values are listed in parenthesis.

**Supplementary Table 1** continued**:**

| Patient group | n (%) | Neuropilin | |  | Follistatin | |  |
| --- | --- | --- | --- | --- | --- | --- | --- |
|  |  | Median | Range | p | Median | Range | p |
| **Disease status** |  |  |  |  |  |  |  |
| EC | 91 (45.0) | 428300 | 234706 - 1110000 | 0.8871 | 1087 | 104.9 - 2926 | 0.3896 |
| Benign | 111 (55.0) | 442973 | 181025 - 846636 | (1.000) | 1185 | 160.8 - 3181 | (0.9483) |
|  |  |  |  |  |  |  |  |
| **Histology** |  |  |  |  |  |  |  |
| Type I | 65 (74.7) | 426242 | 234706 – 1110000 | 0.6798 | 1083 | 104.9 – 2926 | 0.3325 |
| Type II | 22 (25.3) | 466520 | 272268 – 851759 | (0.9989) | 1090 | 293.5 - 1817 | (0.9115) |
|  |  |  |  |  |  |  |  |
| **EC differentiation** | |  |  |  |  |  |  |
| Well differentiated G1 | 46 (61.3) | 426169 | 236598 – 1110000 | 0.5604 | 1098 | 372.8 – 2926 | 0.3013 |
| Moderately differentiated G2 | 19 (25.3) | 400745 | 234706 – 816086 | (0.9928) | 927,0 | 104.9 – 2742 | (0.8837) |
| Poorly differentiated G3 | 10 (13.3) | 467552 | 321741 - 1310000 |  | 982,8 | 514.2 – 1817 |  |
|  |  |  |  |  |  |  |  |
| **FIGO stage** |  |  |  |  |  |  |  |
| IA | 58 (65.9) | 418001 | 236598 – 1020000 | 0.6794 | 1043 | 104.9 – 2923 | 0.9266 |
| IB | 12 (13.6) | 387279 | 237643 – 787523 | (0.9989) | 1132 | 695.4 – 2742 | (1.0000) |
| II | 8 (0.09) | 467962 | 234706 – 1110000 |  | 1062 | 617.3 – 1589 |  |
| III | 6 (0.07) | 509476 | 310264 – 727292 |  | 1102 | 578.1 – 1659 |  |
| IV | 4 (0.05) | 594069 | 324560 – 1310000 |  | 1303 | 514.2 – 1817 |  |
|  |  |  |  |  |  |  |  |
| **Myometrial invasion** |  |  |  |  |  |  |  |
| No invasion | 28 (32.6) | 416024 | 236598 – 1110000 | 0.9581 | 1050 | 293.5 – 2221 | 0.7967 |
| < 50% myometrium | 32 (37.2) | 424183 | 278613 – 1110000 | (1.0000) | 1109 | 104.9 – 2926 | (0.9999) |
| > 50% myometrium | 26 (30.2) | 464123 | 234706 – 851759 |  | 1090 | 514.2 – 1929 |  |
|  |  |  |  |  |  |  |  |
| **Lymphovascular invasion** |  |  |  |  |  |  |  |
| No | 47 (72.3) | 443340 | 236598 – 1190000 | 0.1712 | 1090 | 104.9 – 2926 | 0.6260 |
| Yes | 18 (27.7) | 374101 | 234706 – 851759 | (0.6759) | 1016 | 510.5 – 1888 | (0.9973) |
|  |  |  |  |  |  |  |  |
| **Metastasis** |  |  |  |  |  |  |  |
| No | 78 (88.6) | 424037 | 234706 – 1110000 | 0.4144 | 1075 | 104.9 – 2926 | 0.6762 |
| Yes | 10 (11.4) | 509476 | 310264 – 1310000 | (0.9597) | 1115 | 514.2 – 1817 | (0.9988) |

^a^ p-values were calculated using non-parametric Mann-Whitney tests or Kruskal-Wallis tests with post hoc test and Dunn’s correction. Bonferroni-Šídák method was used for multiple comparison correction and adjusted p-values are listed in parenthesis.

## Single Factor (Univariate) Models

The results obtained on the final ensemble using the ***training*** dataset are shown in Supplementary Table 2. The Threshold Point represents the best offset between the false positive and true positive rate as determined by the ROC curve on the training dataset. The True Negative, False Negative, True Positive and False Positive rates are based on classifying the training samples according to this threshold. The threshold for most univariate models was determined around the middle point (0.5), with the major exception being neuropilin-1. Similarly, the results obtained on the final ensemble using the ***test*** dataset are shown in Supplementary Table 3, where the threshold that was obtained on the training dataset was used. It can be seen that all models perform worse on the test dataset than on the training dataset (higher false negative and false positive rates).

Supplementary Table 2: Thresholds and the corresponding training dataset confusion matrices for models based on seven univariate factors

| Model | Threshold Point | True Negative | False Negative | True Positive | False Positive |
| --- | --- | --- | --- | --- | --- |
| BMI | 0.51949 | 68 | 34 | 41 | 22 |
| Neuropilin-1 | 0.07805 | 26 | 12 | 63 | 64 |
| sTie-2 | 0.45190 | 55 | 22 | 53 | 35 |
| IL-8 | 0.48497 | 72 | 24 | 51 | 18 |
| Follistatin | 0.50465 | 75 | 30 | 45 | 15 |
| Leptin | 0.43751 | 62 | 14 | 61 | 28 |
| G-CSF | 0.38192 | 40 | 34 | 41 | 30 |

Supplementary Table 3: Confusion matrices for all seven trained univariate models on the test dataset

| Model | True Negative | False Negative | True Positive | False Positive |
| --- | --- | --- | --- | --- |
| BMI | 11 | 6 | 10 | 10 |
| Neuropilin-1 | 5 | 8 | 8 | 16 |
| sTie-2 | 10 | 11 | 5 | 11 |
| IL-8 | 11 | 5 | 11 | 10 |
| Follistatin | 15 | 8 | 8 | 6 |
| Leptin | 12 | 2 | 14 | 9 |
| G-CSF | 10 | 4 | 12 | 11 |

Supplementary Table 4: Fisher Exact p-values for confusion matrices of univariate models (significant values are bold)

| Model | p-value for training | p-value for test |
| --- | --- | --- |
| BMI | **< 0.01** | 0.508 |
| Neuropilin-1 | 0.063 | 0.164 |
| sTie-2 | **< 0.01** | 0.316 |
| IL-8 | **< 0.01** | 0.316 |
| Follistatin | **< 0.01** | 0.305 |
| Leptin | **< 0.01** | **0.007** |
| G-CSF | **0.007** | 0.190 |

## Multiple Factor (Multivariate) Models

The results obtained on the final ensemble using the ***training*** dataset are shown in the Supplementary Table 5. They show the threshold point reaching the best balance between false positive and true positive rate and the calculated true positive, true negative, false positive and false negative rates at this threshold. The results obtained on the final ensemble using the ***test*** dataset are then shown in supplementary Table 6. The Threshold Point represents the best offset between the false positive and true positive rate as determined by the ROC curve on the training dataset. The True Negative, False Negative, True Positive and False Positive rates are based on classifying the training samples according to this threshold. The threshold for most multivariate models was determined slightly below the middle point (0.4-0.5), with the major exception of the model combining the AFs without Leptin, where the threshold was determined around 0.34.

Supplementary Table 5: Thresholds and the corresponding confusion matrices for all six trained multivariate models on the training dataset

| Model | Threshold Point | True Negative | False Negative | True Positive | False Positive |
| --- | --- | --- | --- | --- | --- |
| Age+BMI+AFs | 0.46370 | 80 | 12 | 57 | 12 |
| BMI + AFs | 0.50671 | 83 | 9 | 49 | 20 |
| AFs | 0.43142 | 80 | 12 | 57 | 12 |
| BMI + AFs without Leptin | 0.45078 | 76 | 16 | 60 | 9 |
| AFs without Leptin | 0.33593 | 73 | 19 | 58 | 11 |
| Selected Features | 0.459627 | 82 | 10 | 57 | 12 |

Supplementary Table 6: Confusion matrices for all six trained multivariate models on the test dataset

| Model | True Negative | False Negative | True Positive | False Positive |
| --- | --- | --- | --- | --- |
| Age+BMI+AFs | 16 | 3 | 15 | 7 |
| BMI + AFs | 19 | 0 | 9 | 13 |
| AFs | 15 | 4 | 11 | 11 |
| BMI + AFs without Leptin | 17 | 2 | 16 | 6 |
| AFs without Leptin | 16 | 3 | 13 | 9 |
| Selected Features | 15 | 4 | 16 | 6 |

Supplementary Table 7: Fisher Exacts p-values for confusion matrices of multivariate models (significant values are bold)

| Model | p-value for training | p-value for test |
| --- | --- | --- |
| Age+BMI+AFs | **< 0.01** | **< 0.01** |
| BMI + AFs | **< 0.01** | **< 0.01** |
| AFs | **< 0.01** | 0.103 |
| BMI + AFs without Leptin | **< 0.01** | **< 0.01** |
| AFs without Leptin | **< 0.01** | **< 0.01** |
| Selected Features | **< 0.01** | **< 0.01** |

Supplementary Table 8: Pearson correlation coefficients between AFs and Age/BMI (statistically significant correlations marked in bold)

| Variable | Age | BMI |
| --- | --- | --- |
| Age | **1.0000** | 0.0228 |
| BMI | 0.0228 | **1.0000** |
| Neuropilin-1 | 0.1031 | **0.1591** |
| sTie-2 | -0.0421 | 0.0212 |
| IL-8 | **0.2379** | 0.0009 |
| Follistatin | 0.0242 | 0.0973 |
| Leptin | 0.0586 | **0.7751** |
| G-CSF | -0.0063 | **0.1642** |
